# Role of Biofilm in Bacterial Infection and Antimicrobial Resistance

**DOI:** 10.31729/jnma.7580

**Published:** 2022-09-30

**Authors:** Khilasa Pokharel, Bishwa Raj Dawadi, Lok Bahadur Shrestha

**Affiliations:** 1 Department of Microbiology, Kathmandu Medical College and Teaching Hospital, Sinamangal, Kathmandu, Nepal; 2 Department of Emergency Medicine, Grande International Hospital, Dhapasi, Kathmandu, Nepal; 3 School of Medical Sciences and The Kirby Institute, University of New South Wales, Sydney, Australia

**Keywords:** *antibiotic resistance*, *bacterial infections*, *biofilm*, *MRSA*

## Abstract

Biofilm refers to the complex, sessile communities of microbes found either attached to a surface or buried firmly in an extracellular matrix as aggregates. Microbial flora which produces biofilm manifests an altered growth rate and transcribes genes that provide them resistance to antimicrobial and host immune systems. Biofilms protect the invading bacteria against the immune system of the host via impaired activation of phagocytes and the complement system. Biofilm-producing isolates showed greater multidrug resistance than non-biofilm producers. Biofilm causes antibiotic resistance through processes like chromosomally encoded resistant genes, restriction of antibiotics, reduction of growth rate, and host immunity. Biofilm formation is responsible for the development of superbugs like methicillin-resistant *Staphylococcus aureus,* vancomycin-resistant *Staphylococcus aureus,* and metallo-beta-lactamase producing *Pseudomonas aeruginosa.* Regular monitoring of antimicrobial resistance and maintaining hygiene, especially in hospitalized patients are required to control biofilm-related infections in order to prevent antimicrobial resistance.

## INTRODUCTION

Biofilm is commonly seen in chronic conditions and microorganisms remain dormant causing the acute infection.^1,2^ Persistence of bacteria depends upon the formation of biofilm that remains intact on the surface.^3^ When there is a biofilm formation, the host cell develops a fundamental part that forms a biofilm matrix.^4^ This formed matrix inhibits the diffusion of antimicrobials to the cells. This review highlights the structure of biofilm, its effect in gram-positive and negative bacteria, and infections caused by them, and discusses the relation of biofilm with antimicrobial resistance.

## ULTRASTRUCTURE OF BIOFILM

Biofilm is the extracellular polymeric substance (EPS) that provides specific properties during the colonization of bacterial cells.^5,6^ Biofilm is an aggregate of microorganisms with sessile cells, biofouling, corrosion, and deterioration of drinking water quality.^7,8^ Human dental plaque, skin, and gut are dominant biofilm present in Eukaryotes.^9^ Microbial cells inside the biofilm are resistant to physical factors.^10^ EPS accounts for 50% to 90% of the total organic carbon of biofilm.^11^ It is primarily composed of polysaccharides. In the case of Staphylococcus chemical composition of EPS is cationic.^12^ Biofilms are heterogeneous, with microcolonies of bacterial cells enclosed in an EPS matrix and separated from other microcolonies by interstitial voids (water channel).^13^ Biofilm formation can be divided into five stages: initial reversible attachment, irreversible attachment, maturation, and dispersion. The initial contact of the moving planktonic bacteria with the surface is the starting point, which is still reversible at this stage. The bacteria will then start to form a monolayer and will produce an extracellular matrix or slim for protection. The matrix consists of extracellular polysaccharides, structural proteins, cell debris, and nucleic acids; referred to as EPS. The initial steps of the matrix formation are dominated by extracellular DNA (eDNA), whereas polysaccharides and structural proteins take over later.^12,13^ This helps the cells survival, increases the availability of nutrients, and better opportunities for cellular communication and transfer of genetic material among microorganisms which makes them more drug-resistant.

## EPIDEMIOLOGY

Microbial flora which produces biofilm manifests an altered growth rate and transcribes genes that provide them resistance to antimicrobial and host immune systems. Those biofilms contribute to causing chronic inflammatory disease.^14^ From one of the studies that were conducted in 2015, of total gram-positive bacteria and gram-negative bacteria of the total of 190 isolates, 68.9% of isolates showed biofilm formation. Biofilm formation was common in *Pseudomonas aeruginosa, Klebsiella* species, and *Staphylococcus aureus*.^15,16^ It was also detected that biofilm-producing isolates showed greater multidrug resistance than non-biofilm produces.^17-9^ Another study conducted between 2017 to 2018, stated that 40% of the positive isolates from the study were biofilm-producing bacteria of which *Escherichia coli* was found to be the most common organism.^20^

## BIOFILM FORMATION IN GRAM-POSITIVE BACTERIA

Biofilm-associated antimicrobial resistance starts with attachment and gradually increases as biofilm ages.^21^ Effective treatment plans will incorporate antimicrobials and kill biofilm organisms or treatments that disrupt or target specific components of the biofilm matrix.^22^ In Staphylococci, adhesion to human tissue or indwelling devices is regulated by anchored proteins which bind to a host cell, which is referred to as microbial surface components recognizing adhesive matrix molecules.^23^ In *Staphylococci* and *Enterococci,* similar factors contribute to the biofilm matrix composition including polysaccharides, proteins, teichoic acid, lipoteichoic acid, and extracellular DNA.^24^ In *Staphylococci,* the polysaccharide intercellular adhesion (PIA) also known as poly-N-acetyl glucosamine (PNAG), according to chemical composition is an important adhesive molecule during biofilm formation.^25^ From a study done, among gram-positive organisms antimicrobials with different degrees of activity against biofilm, Dalbavancin seems to provide effective therapy in a significant preparation of cases due to its effectiveness in the setting of MDIs (metered-dose inhalers) with a relatively low number of side effects.^26^ From the review it is reflected that biofilm has a clinical impact on the infection and use of potentially therapeutic drugs. The management of several biofilm-related gram-positive infections, it requires prolonged antibiotic therapy.

## BIOFILM FORMATION IN GRAM-NEGATIVE BACTERIA

An increase in drug resistance at both the levels in the community and hospital levels is shown by multidrug resistance and pan resistance which leads to treatment failure increased mortality and morbidity which causes an impact on the cost of medical treatment and prevention of bacterial infectious disease.^27^ Biofilm causes antibiotic resistance through processes like chromosomally encoded resistant gene, restriction of antibiotics, reduction of growth rate, and host immunity.^16^ Knowledge of biofilm formation and antibiogram of bacterial isolates are important for empirical antibiotic therapy.^28^ From one of the studies association between MBL production and biofilm formation was statistically significant whereas it was insignificant between ESBL and biofilm production. Some studies reveal that biofilm and MBL-producing strains were multi-drug resistant.^29^ In another study, antibiotics belonging to the class of Fluoroquinolones, Carbapenems, and Aminoglycosides were found to be more effective for biofilm-producing *Pseudomonas aeruginosa, Escherichia coli,* and Klebsiella pneumoniae. Understanding biofilm will help in the novel treatment and will reduce real threat.^30^ Biofilm is also a major virulence factor in the case of Uropathogenic *Escherichia coli*.^31^ From the studies it is revealed that the major problem of biofilm formation is its battle with antimicrobial agents. Mechanical control like avoidance of attachment of bacteria to surfaces, creating a smooth surface, hydrophobicity, and degradation of formed biofilms can be done for the avoidance of biofilm formation. Physical control measures like super high magnetic fields, ultrasound treatment, etc can be used to control biofilm formation.

## ROLE OF BIOFILM PRODUCTION CAUSING METHICILLIN AND VANCOMYCIN RESISTANCE

Vancomycin has long been considered the last resort treatment for Methicillin-resistant *Staphylococcus aureus* (MRSA) infection.^32^ Its excessive use resulted in the emergence of Vancomycin-intermediate *Staphylococcus aureus* (VISA), Vancomycin-resistant *Staphylococcus aureus* (VRSA), and heterogeneous Vancomycin intermediate *Staphylococcus aureus* (hVISA) strains.^33^ MRSA strains are able to form biofilm as a fitness and survival mechanism that is mediated by strong adhesion, increase in drug resistance, and reduction of effectiveness of sanitizers. In presence of biofilm, the resistance of *Staphylococcus aureus* to antimicrobials was reported to increase by 1000 times.^34^ Ability of MRSA and VRSA isolates to produce biofilms and the presence of high rates of antimicrobial resistance is quite alarming.^35^

## ROLE OF BIOFILM PRODUCTION CAUSING CARBAPENEM RESISTANCE

The biofilm-forming ability of most gram-negative bacteria is high. In one of the phenotypic studies, it has been shown that there is a positive correlation between biofilm formation and carbapenem resistance.^36^ From the same study it has been mentioned that strong biofilm reduces the number of days alive for the patient to 3.33 days from poor or negative biofilm- producing isolates with 11.33 days. It demonstrates that the production of biofilm increases the mortality rate. In one of the studies done biofilm formation in Carbapenem susceptible and carbapenem-resistant *Pseudomonas aeruginosa isolates* is 83.6% and 95.0% respectively.^37^

## KNOWLEDGE GAP ON BIOFILM

Host tissue-related biofilm infections are often chronic, including chronic lung infections of cystic fibrosis patients, chronic osteomyelitis, chronic prostatitis, chronic rhinosinusitis, chronic otitis media, chronic wounds, recurrent urinary tract infection, endocarditis, periodontitis, and dental caries.^9,10,15^ The ability of pathogenic biofilms to survive in presence of a high concentration of antibiotics is called recalcitrance leads to treatment failure, infection recurrence, and chronic infections.^9^ The exact extent to which antimicrobial resistance contributes to antimicrobial resistance is still not crystal clear.^10^ Recently, some research regarding biofilm formation and antimicrobial resistance are being conducted in Nepal. To our knowledge, there is a lack of a good review article summarizing research from Nepal, which we are attempting to address. This review article will be helpful in informing the clinicians and nurses regarding the current scenario of biofilm formation and the importance of good infection control measures.

## INFECTION CAUSED BY BIOFILM

Biofilm formation of infectious significance is found in implant devices. *Pseudomonas aeruginosa* is the second most common cause of ventilator-associated pneumonia (VAP) and Catheter-associated urinary tract infection (CAUTI).^38,39^ *Pseudomonas aeruginosa* forms biofilms on endocardial tubes and catheters in CAUTI and VAP patients.^40^ Mostly biofilms occur with indwelling medical devices such as Central venous Catheters, peritoneal dialysis catheters, mechanical heart valves, and urinary catheters.^41-4^ Biofilm composed depends on devices and the duration of action of microbial species. Microorganism causing periodontitis, like *Pseudomonas aerobicus* and *Fusobacterium nucieatum* has the ability to form biofilm on the mucosal surface in the oral cavity.^45^ Microbial colonization of teeth surface permits them to invade mucosal cells and alter the flow of calcium. In epithelial cells they release toxins. Plaque then develops within 2-3 weeks. Plaque then mineralizes with calcium and phosphate ions which form calculus.^46-8^ Biofilm formation on medical devices affects surgical and instrumental procedures and public health. Biofilm formation also has implications for non'device'related health complications. Therefore, good hygiene conditions and practices are necessary to avoid biofilm formation.^49-52^

## WAY FORWARD

This review was made to identify and characterize biofilm, determine the biofilm-forming bacteria, and examine its effect on the pathogenesis of bacterial infection and antimicrobial resistance. In this study, we have summarized the relationship between biofilms and the extent of antimicrobial resistance. So, we need to prevent biofilm formation through strict antimicrobial resistance surveillance, proper hygiene, and proper infection control measures, especially during the implantation of an intravenous catheter, mechanical ventilation, and urinary catheterization. Regular detection of biofilm formation by various phenotypic and molecular methods should be introduced in all health care settings.
